# Cancer-associated fibroblasts and their influence on tumor immunity and immunotherapy

**DOI:** 10.7554/eLife.57243

**Published:** 2020-12-28

**Authors:** Richard Lee Barrett, Ellen Puré

**Affiliations:** University of PennsylvaniaPhiladelphiaUnited States; University of Paris DescartesFrance; PfizerUnited States

**Keywords:** immunotherapy, fibroblast, cancer, tumor immunity, T-cell, macrophages

## Abstract

Fibroblasts play an essential role in organogenesis and the integrity of tissue architecture and function. Growth in most solid tumors is dependent upon remodeling ‘stroma’, composed of cancer-associated fibroblasts (CAFs) and extracellular matrix (ECM), which plays a critical role in tumor initiation, progression, metastasis, and therapeutic resistance. Recent studies have clearly established that the potent immunosuppressive activity of stroma is a major mechanism by which stroma can promote tumor progression and confer resistance to immune-based therapies. Herein, we review recent advances in identifying the stroma-dependent mechanisms that regulate cancer-associated inflammation and antitumor immunity, in particular, the interactions between fibroblasts and immune cells. We also review the potential mechanisms by which stroma can confer resistance to immune-based therapies for solid tumors and current advancements in stroma-targeted therapies.

## Introduction

Cancer cells must construct an extensive network of local and distant communications in order to establish a ‘neo-organ’ system that provides the connective tissue cells, extracellular matrix (ECM), vasculature, and immune privilege required to sustain tumor growth. While traditionally, therapies were designed to target the cancer cells directly, recent complementary efforts are aimed at disrupting such networks that support malignant cell behavior and are proving to be a successful therapeutic space. Examples include anti-angiogenic drugs (Jayson et al., 2016; Al-Husein et al., 2012) and immunotherapies that are rapidly changing the therapeutic landscape for a wide variety of cancers. Moreover, cancer-associated fibroblasts (CAFs) are emerging as potential therapeutic targets in the rapidly expanding field of stroma-targeted therapies designed to complement malignant cell-targeted therapies.

Immunotherapies are proving particularly effective in the clinic and are rapidly being incorporated into standards of care, alongside chemotherapy, for a variety of cancer types. Immunotherapy can be broken down into two general categories. The first are therapies designed to activate or amplify the immune system through small molecules, biologics, or antibodies. Such therapies include cancer vaccines (Melief et al., 2015), immune agonists such as anti-CD40 (Beatty et al., 2017), and inhibitors of immune checkpoints such as anti-CTLA-4 (Leach et al., 1996) and anti-PD-1/PD-L1(Iwai et al., 2002; Gong et al., 2018) antibodies. The second is based on adaptive transfer of immune cells such as T cells expressing tumor antigen reactive T cell receptors (TCR) and chimeric antigen receptor (CAR) expressing T cells (CAR-T) (June et al., 2018; Holzinger et al., 2016; Rosenberg et al., 2008). However, while effective in some patients, substantial proportions of patients are resistant to or acquire resistance to immune modulators. Moreover, although CAR-T cell therapy has been particularly effective in hematologic tumors (Maus et al., 2014), little success has been realized to date in solid tumors with this approach. Such resistance highlights the need for better understanding of how tumor immunity is regulated in the tumor microenvironment (TME).

Although their functionality has been largely overlooked in the context of cancer, fibroblasts—and their associated matrix—account for the majority of tumor mass in many cancer types (Marsh et al., 2013) and fibroblasts are known to influence malignant cells behavior through both biochemical and biomechanical signals. Additionally, fibroblasts can exert a strong immunomodulatory influence, regulating the infiltration and phenotype of immune cells within the TME and influencing their spatial localization and functionality intra-tumorally. In this review, we focus on the intersection of fibroblast-derived factors and tumor immunity and discuss the role of fibroblasts in immunotherapy resistance.

### Fibroblast activation

Fibroblasts are a difficult cell type to define due to a lack of unique markers expressed exclusively and by all fibroblasts (Sahai et al., 2020). Fibroblasts are heterogeneous populations of mesenchymal cells that perform essential roles in formation and maintenance of connective tissue ECM and govern parenchymal cell behavior. Fibroblasts are found in virtually all tissues and, under homeostatic conditions, exist in a quiescent state defined by a low proliferative capacity and metabolic state. Fibroblasts, like resident leukocytes, serve as sentinels that sense disruptions in homeostasis, and fibroblast activation is a common early response to such disruptions. Activation is characterized by increased proliferative capacity, increased synthetic activity—including production of a provisional matrix and production of growth factors, cytokines, and chemokines—and increased metabolic activity, all designed to restore homeostasis (Darby and Hewitson, 2007; Darby et al., 2014). This reparative fibro-proliferative response is arguably best understood in the context of wound healing, where specialized subsets of activated fibroblasts proliferate and migrate into the wound site, produce ECM to restore damaged tissue, and act in concert with inflammatory, immune, and other cells as necessary to restore homeostasis (Darby and Hewitson, 2007; Darby et al., 2014). However, many features of fibroblast activation are shared in other contexts, including cancer, where activated CAFs can strongly influence the TME. Moreover, both quiescent and activated fibroblasts are heterogeneous and exhibit marked context-dependent diversity in their phenotypes and functionality.

**Figure 1.**
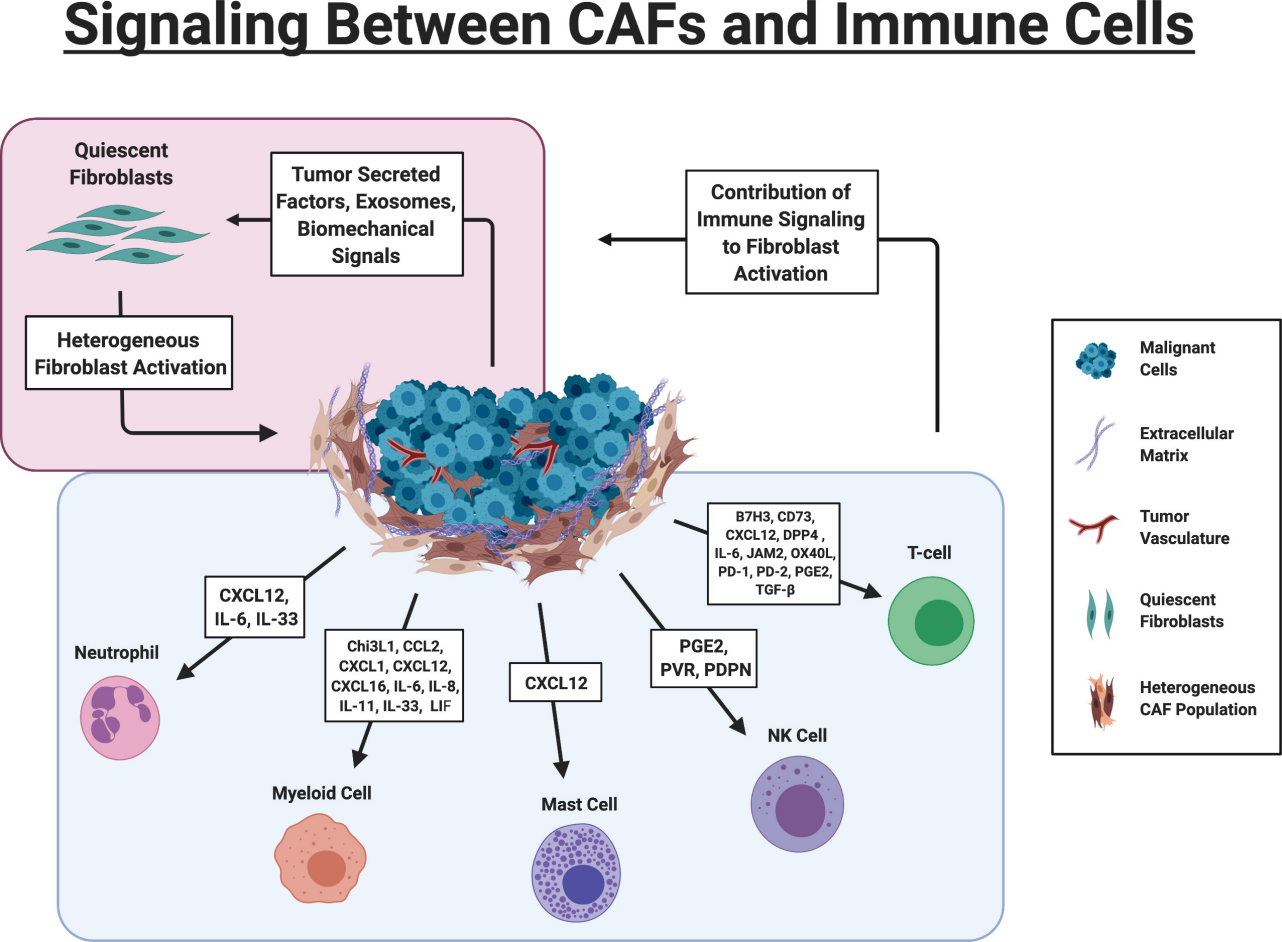
Signaling Between CAFs and Immune Cells. Red Box: Tumor cells drive activation of fibroblasts via multiple mechanisms. Fibroblasts are heterogeneously activated into different sub-populations based on the biochemical and biomechanical cues in their immediate environment. Blue Box. Key signaling pathways involved in CAF-immune cell signaling. B7H3 (CD276); 5′-nucleotidase (CD73); Chitinase 3-like 1 (Chi3L1); chemokine (C-C motif) ligand 2 (CCL2; chemokine (C-X-C motif) ligand 1 (CXCL1)); chemokine (C-X-C motif) ligand 12 (CXCL12), chemokine (C-X-C motif) ligand 16 (CXCL16), Dipeptidyl peptidase 4 (DPP4); Junctional adhesion molecule B (JAM2); Interleukin 6 (IL-6); Interleukin 8 (IL-8); Interleukin 11 (IL-11); Leukemia Inhibitory Factor (LIF), tumor necrosis factor receptor superfamily member four ligand (OX40L); Programmed cell death protein 1 (PD-1), Programmed cell death protein 2 (PD-2), Prostaglandin E2 (PGE2), Transforming growth factor beta (TGF-β), Polio Virus Receptor (PVR), CAF) (Figure created with BioRender.com).

Understanding the relationship between tumor cells, tumor infiltrating immune cells, and CAFs has been hampered by an incomplete understanding of CAF biology such as the intrinsic and extrinsic factors involved in their activation. The mechanisms underlying the phenotypic and functional heterogeneity, spatiotemporally dynamic evolution, and plasticity of CAFs are also poorly understood. Cancer cells can drive fibroblast activation through both biochemical and biomechanical mechanisms (Figure 1). Tumor cell secreted factors (for example, TGF-β, PDGF, EGF, CTGF, and FGF [Varga and Jimenez, 1986; Gardner et al., 2004; Postlethwaite et al., 1987; Montesano and Orci, 1988; Kuzet and Gaggioli, 2016; Löhr et al., 2001; Takehara, 2000]), and tumor cell-derived exosomes, have been implicated in fibroblast activation (Ringuette Goulet et al., 2018). As with classical wound healing, CAFs can also become activated in response to damage-associated molecular patterns (DAMPs) released by damaged tissue or necrotic tumor cells. These signals are sensed by fibroblasts through pathways such as the NLRP3 inflammasome (Ershaid et al., 2019).

In addition to malignant cells, endothelial, inflammatory and immune cells in the TME also secrete mediators of fibroblast activation. Moreover, activated fibroblasts produce factors and matrix (Kojima et al., 2010) that drive their autocrine activation in a feed-forward manner, as fibroblasts are responsive to substratum composition and stiffness (Avery et al., 2018; Hadjipanayi et al., 2009). Stiffness within compliant normal tissues typically ranges from ~0.05 to 5kPa (Cox and Erler, 2011), while progressive stiffening of tumor tissue can reach up to ~20 kPa in the most desmoplastic tumors such as pancreatic cancer (Kalli and Stylianopoulos, 2018). Moreover, substratum composition and stiffness may also be important factors in driving heterogeneity amongst activated fibroblasts (Avery et al., 2018). Integration of biochemical and biomechanical signals can also drive dedifferentiation of other mesenchymal cell types, such as pericytes and adipocytes, to a CAF-like state (Hosaka et al., 2016; Nieman et al., 2013; Zoico et al., 2016). Notably, diverse cancer types and tumors at different stages of progression, can vary with regard to prevalence of, and dependence on stroma.

### Activated fibroblasts are heterogeneous

In view of clinical evidence for a correlation between CAF markers and poor overall prognosis in multiple cancer types, CAFs were conventionally considered to be pro-tumorigenic (Calon et al., 2015; Comito et al., 2014). However, initial attempts at depleting myofibroblasts from the TME unexpectedly enhanced tumor progression (Özdemir et al., 2015; Rhim et al., 2014). Such seemingly confounding observations led to the current push to better understand the heterogeneity of fibroblasts within the TME.

Historically, it was common to rely on expression of alpha-smooth muscle actin (α-SMA) as a hallmark of activated fibroblasts. These fibroblasts were generically referred to as ‘myofibroblasts’. Myofibroblasts were originally defined in the context of wound healing where their expression of components of the contractile apparatus typical of smooth muscle cells—including α-SMA—confers cells with the contractile function critical for wound closure and contribute to tissue tension (Darby and Hewitson, 2007; Darby et al., 2014). Myofibroblasts are indeed common to multiple physiologic reparative responses and also to pathologic conditions such as scarring, tissue fibrosis, and to solid tumors that are characterized by a sustained pathogenic fibro-proliferative response (Phan, 2002; LeBleu et al., 2013; Hinz et al., 2007). However, myofibroblasts represent only a subset of activated fibroblasts within the TME.

CAFs are in fact heterogeneous based on phenotypic markers, gene expression profiling, and functionality (Helms et al., 2020). So far, a pan-specific CAF marker has yet to be identified and neither a unifying approach to defining, nor a standardized nomenclature for CAF subpopulations has yet emerged. Defining CAF subsets promises to be complex based on evidence that the state of fibroblast activation is both context-dependent and plastic.

Further adding to the complexity is that fibroblasts in healthy tissue, from skin to pancreas, exhibit heterogeneity even before activation (Philippeos et al., 2018; Garcia et al., 2020). Understanding this underlying heterogeneity is vital to understanding the development of CAFs within the TME. For example, two mostly distinct populations comprise the local fibroblasts in healthy pancreas—identified as Gli1^+^ and Hoxb6^+^, respectively—however, lineage-tracing studies demonstrated that these two populations do not contribute equally to CAF development in pancreatic cancer. Specifically, the Gli1^+^ population was found to expand significantly during carcinoma development, while the more stellate cell-like Hoxb6^+^ fibroblasts did not appear to contribute to the same degree (Garcia et al., 2020). Additional lineage-tracing experiments will provide critical insights and facilitate future understanding into how resident fibroblast populations might differentially contribute to CAF development.

In established tumors, recent single-cell sequencing analyses indicate that fibroblasts segregate into anywhere from three to seven major clusters based on transcriptome (Elyada et al., 2019; Lambrechts et al., 2018; Dominguez et al., 2020). However, it is premature to make conclusions regarding the extent to which multiple subpopulations—being defined by gene signatures based on single cell RNAseq data—may overlap between diverse fibroinflammatory settings or even between CAFs described in different types of carcinomas. Current evidence taken together does however suggest that there is considerable context-dependent variability in definable CAF clusters between tumor types and also between TME and other fibroinflammatory conditions. Further analysis across tumor types and other fibroinflammatory settings will be required to reveal any potentially unifying patterns of subpopulations of activated fibroblasts in diverse pathophysiologic settings. Nonetheless, some patterns are emerging within particular tumor types, and there is now some evidence for shared subpopulations of CAFs defined by phenotype or gene expression signatures between tumor types as discussed below.

Two broadly defined subpopulations referred to as myCAFs and iCAFs, were first described in pancreatic cancer (Sahai et al., 2020; Elyada et al., 2019; Öhlund et al., 2017; Biffi et al., 2019). Single-cell analysis of stroma provided evidence of similar populations in other cancer types, such as lung (Lambrechts et al., 2018) and colorectal cancer (CRC) (Li et al., 2017).

MyCAF encompass the traditional myofibroblast population mentioned earlier. MyCAFs tend to be enriched amongst the most tumor adjacent fibroblasts where tumor secreted factors such as TGF-β, and microenvironmental cues such as increased tissue stiffness, drive and sustain the phenotype (Avery et al., 2018; Midgley et al., 2013). MyCAFs are hyperproliferative compared to other CAF populations and in certain contexts may be tumor restraining (Özdemir et al., 2015; Rhim et al., 2014). iCAF refers to an inflammatory CAF phenotype, defined by expression of cytokines such as IL-6 and CXCL12, and commonly identified by expression of Ly6C (Elyada et al., 2019; Öhlund et al., 2017; Biffi et al., 2019). Ly6C is also a marker of inflammatory macrophages raising the interesting possibility of a common transcriptional program regulating inflammatory genes shared by multiple cell types that contribute to the inflammatory milieu in the TME. The iCAF population tends to be more distal from the edge of tumor cell nests and is driven by tumor secreted factors such as IL-1(*46, 47*). IL-1 signaling can drive expression of autocrine signals in iCAFs such as LIF, which help to maintain their inflammatory phenotype. These two populations are interconvertible with evidence suggesting that myCAFs may dominate functionally in at least some instances where both populations are present (Öhlund et al., 2017; Biffi et al., 2019). Studies highlighting these two populations in pancreatic cancer have also described a third population defined by their expression of MHC-II and thus described as antigen-presenting CAFs (apCAFs) (Elyada et al., 2019). The variable influence of these different CAF populations on tumor immunity is further discussed throughout this review.

Determining whether iCAF and myCAF designations based on these criteria will apply broadly awaits single-cell RNA sequencing of additional tumor types and validation at the protein and functional levels. Further studies will also be required to relate these designations to other phenotypically and functionally defined subpopulations.

Independently, a number of other markers defining activated fibroblast subpopulations have been noted. These include CD29 (Costa et al., 2018), CD90 (Huynh et al., 2016), caveolin (CAV1) (Costa et al., 2018), fibroblast activation protein (FAP) (Park et al., 1999), podoplanin (PDPN) (Kitano, 2010; Astarita et al., 2012), fibroblast-specific protein 1 (FSP-1/S100A4) (Sugimoto et al., 2006; Strutz et al., 1995), meflin (Maeda et al., 2016), and platelet-derived growth factor receptor alpha (PDGFRα) (Sugimoto et al., 2006; Farahani and Xaymardan, 2015; Nurmik et al., 2020; Table 1). These markers can be used alone or combinatorically to distinguish subpopulations with key functional differences within the TME (see Table). For example, FAP expressing fibroblasts can be further divided based on functionality and/or the presence of other markers such as α-SMA, while PDPN^+^ cells can be subdivided by their expression of FAP, with the FAP^-^PDPN^+^ cells being identified as pericytes for instance (Table 1; Cremasco et al., 2018).

**Table 1.** Common markers recently used to study CAF populations that influence tumor immunity and progression. Many potential markers have been described throughout the literature; however, this table has been limited to the markers most relevant to the topics outlined in this review.

Phenotypic marker	Features of expressing populations	Reported tumor types	Subcellular localization	Refs
aSMA (ACTA2)	Myofibroblasts/myCAFs Context dependent tumor promotion and/or tumor restraint Preferentially located tumor adjacent Contractile	Most	Cytoplasmic	Özdemir et al., 2015; Costa et al., 2018; Zhou et al., 2018; Kato et al., 2018
FAP (FAP)	Tumor promoting through immune-dependent and immune-independent mechanisms Major producers of immunosuppressive cytokines like CXCL12 and CCL2 in the TME	Most	Membrane	Feig et al., 2013; Yang et al., 2016; Lo et al., 2015; Lee et al., 2011
FSP1 (S100A4)	Commonly used fibroblast marker Marks both quiescent and activated fibroblasts Also present on macrophages	Most	Cytoplasmic, Nuclear	Strutz et al., 1995; Österreicher et al., 2011
Gli1 (GLI1)	Fibroblast sub-population that closely associates with vasculature and ducts in pancreas Preferentially expands over other fibroblast populations during pancreatic tumor progression	Pancreatic	Cytoplasmic, Nuclear	Garcia et al., 2020
Hoxb6 (HOXB6)	Fibroblast sub-population present dispersed throughout healthy pancreas Minimal contribution to desmoplasia in pancreatic tumor progression	Pancreatic	Nuclear	Garcia et al., 2020
LRRC15 (LRRC15)	TGF-β-driven gene expression signature Correlate with poor tumor immunity	Pancreatic	Membrane	Dominguez et al., 2020
Ly6C (Ly6c1)	Defines iCAF population in mouse, in combination with other CAF markers Common on myeloid cells	Pancreatic	Membrane	Elyada et al., 2019; Biffi et al., 2019
Meflin (ISLR)	Suppress PDAC progression Expression correlates with CD8^+^ T cells, macrophages and dendritic cells in gastric cancer	Pancreatic, gastric	GPI-linked Membrane Protein	Maeda et al., 2016; Mizutani et al., 2019; Li et al., 2020
PDGFRα (PDGFRA)	Pan-fibroblast marker Marks both quiescent fibroblasts and CAFs	Most	Membrane	Farahani and Xaymardan, 2015; Nurmik et al., 2020
PDPN (PDPN)	Associated with poor tumor immunity Marker of follicular RCs and some macrophages	Most	Membrane	Kitano, 2010; Astarita et al., 2012; Shindo et al., 2013; Kerrigan et al., 2012

## CAF-immune cell interactions

CAFs are now understood to be a major source of immunosuppressive activity in the TME (Figure 1). Fibroblasts can influence immune cell infiltration either directly—via secreted cytokines and chemokines and cell surface proteins—or indirectly—through deposition of various ECM components and remodeling of matrix on which immune cells depend for intra-tumoral localization and migration. Better defining these interactions is particularly important in the context of expanding immunotherapies, as CAFs can not only influence de novo immune responses but dictate the success of immunotherapies as well. Notably, many studies of CAF-immune cell interactions to-date were performed using heterogeneous CAF populations and therefore, specific observations may be manifestations of either a balance between multiple subpopulations or of a predominant subpopulation present. Importantly, the representation of distinct subpopulations may vary dramatically depending on in vivo context.

Although remodeling of ECM by tumor cells, inflammatory cells, and CAFs is another important mechanism by which immune cell recruitment and function are regulated in the TME, in the interest of space and focus, in this review, we focus our discussion in particular on direct signaling between CAFs and immune cells in the context of cancer and refer the reader to other recent reviews that focus on ECM influences on tumor immunity (Sorokin, 2010; Kai et al., 2019; Yamauchi et al., 2020).

### Myeloid cells

Myeloid cells are well studied in the context of the TME where they exhibit remarkable phenotypic plasticity (Kiss et al., 2018), but often acquire an immunosuppressive state that suppresses cytotoxic T cell activity and subdue anti-tumor immunity (Gabrilovich and Nagaraj, 2009). Early evidence that CAFs may contribute to this immunosuppressive phenotype was based on clinical data showing a correlation between stromal markers and immunosuppressive cell types such as tumor-associated macrophages (TAMs) and myeloid-derived suppressor cells (MDSC) (Zhou et al., 2018; Takahashi et al., 2017; Herrera et al., 2013). The development of MDSCs correlates not only with poorer overall survival across a variety of cancers, but also with whether or not patients will respond to immunotherapy (Calon et al., 2015; Diaz-Montero et al., 2014).

CAFs can secrete a number of factors known to influence both recruitment and activation state of myeloid cells including: Chi3L1, CXCL1, CXCL2, CXCL5, CXCL6/GCP-2, CXCL8, CXCL9, CXCL10, CXCL16, CXCL12/SDF1, CCL2/MCP-1, CCL3, CCL5/Rantes, CCL7, CCL20, CCL26, IL-1β, IL-6, IL-8, IL-10, leukemia inhibitory factor (LIF), VEGF, TGF-β, indoleamine-2,3-dioxygenase (IDO), prostaglandin (PG) E2 (PGE2), tumor necrosis factor (TNF), or nitric oxide (NO) (Ziani et al., 2018; Lazennec and Richmond, 2010). To date, only a handful have been directly implicated in CAF-immune crosstalk within the TME (Figure 1).

Two of the more well-studied pathways in CAF-myeloid interactions are CXCL12/CXCR4 and IL-6/STAT3. CAFs are the primary source of CXCL12 in the TME (Feig et al., 2013). CXCL12 recruits myeloid cells to the TME while inducing an immunosuppressive phenotype. Inhibiting either CXCL12 or its receptor CXCR4 is sufficient to decrease intra-tumoral MDSCs and improve overall immune response to a variety of cancers (Comito et al., 2014; Feig et al., 2013; Benedicto et al., 2018; Obermajer et al., 2011; Gok Yavuz et al., 2019; Deng et al., 2017). Expression of CXCL12/CXCR4 is regulated by PGE2 and TGF-β and these have been proposed as potential upstream targets for inhibiting this signaling axis (Kojima et al., 2010; Obermajer et al., 2011; Katoh et al., 2010).

The IL-6/STAT3 pathway has a similar effect. CAF-derived IL-6 drives STAT3 activation in myeloid cells, driving them to more immunosuppressive cell types such as regulatory dendritic cells (DCs) (Cheng et al., 2016). Blocking either CAF IL-6 or myeloid STAT3 can disrupt signaling from CAFs to myeloid cells and attenuate immunosuppression in hepatocellular carcinoma (HCC) and pancreatic cancer (Deng et al., 2017; Chomarat et al., 2000; Mace et al., 2013).

Also within the IL-6 family of cytokines, LIF and IL-11 have been identified as major mediators of CAF paracrine signaling and STAT3 activation in the TME (Shi et al., 2019; Heichler et al., 2020). LIF signaling from CAFs drives immunosuppressive gene expression in TAMs that includes upregulation of CCL2—an important signal for myeloid cell attraction to the TME—and silencing of CXCL9—a cytokine which drives recruitment of cytotoxic T-cells (Pascual-García, 2019). While CAFs can elicit CCL2 expression from TAMs via this LIF-mediated paracrine signaling, CAFs—in particular FAP^+^ CAFs—are capable of producing CCL2 directly (Yang et al., 2016). These FAP^+^ CAFs are also known to be the main source of CXCL12 in the TME (Feig et al., 2013). Such expression patterns bring up an important concept in regard to CAF heterogeneity that, while many CAF signaling pathways are often described at a whole population level, many specific cytokines can be traced back to specific CAF subpopulations.

Other myeloid targeting cytokines produced by CAFs include CXCL16 and IL-33—both important for myeloid cell recruitment (Allaoui et al., 2016; Shani et al., 2020), chitinase 3-like 1 (Chi3L1)—which drives M2 polarization in macrophages (Cohen et al., 2017), and CXCL1—which has been implicated in CAF-mediated myeloid suppressor cell accumulation with important considerations in therapy as discussed below (Kumar et al., 2017). However, which CAF signaling pathways are the predominant drivers of myeloid phenotype are context-dependent as, studies showing the role of IL-8/CXCR2 in the recruitment of myeloid cells in CRC were associated with little, if any, contribution from CXCL12/CXCR4 (Chen et al., 2019b; Zhang et al., 2019). The ratio of different fibroblast subpopulations and influence of the specific cancer type likely play a role in determining the dominate signaling pathways.

### T cells

Given that T cells are prominent cytotoxic responders to cancer cells, some of the most extensive work on CAFs’ role in tumor immunity have been done in this space. Understanding these interactions is of compounded importance as many of the barriers to efficacy in T-cell-based therapies, such as checkpoint blockade and adoptive T cell therapy like CAR-T, are thought to be a result of these immunosuppressive stromal interactions.

As with myeloid cells, CAF markers reportedly correlate with immunosuppression of T cells in the TME, as defined by an increased ratio of FoxP3^+^ T cells over CD8^+^ T cells, which was also associated with poor clinical outcome (Kato et al., 2018; Kinoshita et al., 2013). While this regulation of the adaptive immune response could be explained by CAFs’ effects on myeloid cells, CAFs can also act on T cells directly by promoting regulatory T cells and blocking the effects of cytotoxic T cells (Ziani et al., 2018; Figure 1).

The role of CAFs in driving regulatory T cell responses is supported by experiments showing that flank tumors co-injected with fibroblasts show a greater ratio of FoxP3^+^ to CD8^+^ T cells than tumor cells injected alone. This effect is thought to be mediated via CAF secreted IL-6 as, anti-IL-6 antibodies attenuated the immune effect in vivo (Kato et al., 2018). Further, fibroblast IL-6 drives differentiation of other regulatory T cells such as interleukin-17 (IL-17)-producing T helper (T_H_17) cells (Komatsu et al., 2014). T_H_17 cells exhibit both pro- and anti-tumorigenic effects depending on the tumor type. On one side, T_H_17 cells can recruit CD8^+^ T cells to the TME helping to drive antitumor immune response (Martin-Orozco et al., 2009); on the other, IL-17 released from T_H_17 cells can in turn, drive production of angiogenic factors from fibroblasts and cancer cells (Numasaki, 2003), thus fueling tumor growth. The role of T_H_17 cells varies between cancer types and is likely determined by the TME and further signaling from CAFs (Guéry and Hugues, 2015).

T cells also interact with a number of cell surface ligands displayed by CAFs including B7H3, CD73, DPP4, JAM2, and OX40L (Costa et al., 2018; Figure 1). CAF expression of JAM2, OX40L, and PD-L2, significantly increases the duration of contact time between T cells and CAFs, while B7H3, CD73, and DPP4 play a more direct role in allowing CAFs to drive expansion of CD25+FOXP3+ T cell numbers (Costa et al., 2018; MacGregor et al., 2019; Yu, 2020). B7H3 is an immune checkpoint molecule (Castellanos et al., 2017), CD73 converts AMP to immunosuppressive adenosine (Beavis et al., 2012), and DPP4 cleaves the proinflammatory cytokine CXCL10 to a truncated version that acts as an antagonist to its own receptor CXCR3(*101*). All three targets are expressed by cells other than fibroblasts within the TME, including cancer cells themselves, so the extent to which CAF-specific expression influences the TME may vary. Still, potential immunotherapies are developing around each target with success in early preclinical models (Barreira da Silva et al., 2015; Chen et al., 2019b; Yang et al., 2020).

CAFs can also influence T cell immunity through antigen presentation. CAFs are capable of sampling, processing, and presenting tumor antigens via major histocompatibility complex 1 (MHC-I). T cells engage the MHC-I-antigen bimolecular complex along with PD-L2 and FASL on the CAF cell surface. The combined signaling can result in killing of antigen-specific cytotoxic CD8 T cells (Lakins et al., 2018). In addition to MHC-I-dependent antigen presentation, a population of MHC-II expressing fibroblasts, mentioned earlier in the review as apCAFs, have also been described in pancreatic cancer. However, unlike professional antigen presenting cells, apCAFs express PGE_2_ and lack co-stimulatory ligands necessary to activate immune cells so that MHC-II signaling apCAFs promotes expansion of CD4^+^, CD25^high^, Foxp3^+^ regulatory T cells (Tregs) (Elyada et al., 2019; Pinchuk et al., 2011) rather than activation of effector T cells with potential to mediate antitumor immunity. It should be noted that detection of apCAFs is variable, even within the pancreatic TME in which they have been described (Dominguez et al., 2020).

PD-L1 and PD-L2 are important signaling ligands expressed by many cell types to limit T cell function and prevent autoimmunity (Barclay et al., 2018). Consequently, PD-L1/2 expression in the TME promotes immunosuppression, protecting cancer cells from T cell-mediated killing, and thus has become an important target for checkpoint inhibition. Fibroblasts within the TME can express PD-L1/2 themselves (Pinchuk et al., 2008; Nazareth et al., 2007; Cho et al., 2011) as well as increase PD-L1/2 expression in tumor cells via CXCL5 signaling (Li et al., 2019)—as shown in melanoma and CRC—or miR-92 delivered via CAF-derived exosomes—as shown in breast cancer (Dou et al., 2020).

Even when T cells are functionally capable of mounting a response against tumor cells, as would be the case in the presence of checkpoint inhibitors or in the case of adoptively transferred CAR-T cells, the stroma consisting of CAFs and remodeled ECM, can act as a formidable barrier to T cells, resulting in T cell exclusion from tumor nests (Ford et al., 2020). Expression of TGF-β-associated ECM genes in CAFs is one of the strongest predictors of immunotherapeutic failure, and co-administration of anti-TGF-β antibodies and anti-PD-L1 antibodies significantly improved success of immunotherapy in preclinical models (Mariathasan et al., 2018; Chakravarthy et al., 2018). Inhibiting TGF-β renders solid tumors more T cell responsive and susceptible to therapies such as anti-PD-1-PD-L1 therapy (Tauriello et al., 2018). Specific downstream targets of TGF-β that contribute to this effect include NOX4, and inhibition of NOX4 makes tumors more susceptible to immunotherapy (Ford et al., 2020). The CXCL12/CXCR4 pathway also drives T cell exclusion and inhibition of CXCL12 with the clinical-stage l-RNA-aptamer NOX-A12 also improved response to anti-PD-1 therapy (Zboralski et al., 2017).

Not all fibroblast-mediated signaling in the TME is immunosuppressive however, and a number of studies highlight fibroblast populations capable of stimulating cytotoxic T cell responses. In some settings, IL-6 signaling from fibroblasts exposed to T cells enhanced T cell stimulation (Barnas et al., 2010). Such data highlights the functional and clinical significance of CAF heterogeneity in vivo with certain populations performing opposing functions and exhibiting distinct secretomes (Nazareth et al., 2007). It is worth noting, that immune suppressive fibroblast populations may even be over-represented in past literature, as common in vitro methods of cultivating fibroblasts can promote transition of fibroblasts into an immunosuppressive state, artificially minimizing the potential contribution of other populations (Barnas et al., 2010; Ghebeh and Dermime, 2007).

### Natural killer cells

Our understanding of the role of natural killer (NK) cells in tumor immunity has grown considerably in recent years (Nicholson et al., 2019; Ben-Shmuel et al., 2020). Like T cells, NK cells possess the intrinsic ability to detect and kill malignant cells (Nicholson et al., 2019). However, also similar to T cells, NK cell responses are greatly influenced by CAFs in the TME which can interfere with NK cell-mediated killing (Bassani et al., 2019).

In co-culture, CAFs interfere with key processes in NK cell activation including IL-2-mediated up-regulation of triggering receptors NKp44, NKp30, and DNAM-1 and acquisition of cytolytic granules. While DNAM-1 inhibition appears to be contact-dependent with CAFs, inhibition of NKp44 and NKp30 expression is driven by CAF secretion of PGE_2_(*122*). Also contact-dependent, expression of podoplanin in certain fibroblast subpopulations can interfere with CCL21/CCR7-directed adhesion and migration of NK cells in the TME (Tejchman et al., 2017; Figure 1).

PGE_2_ is a major CAF-derived inhibitor of NK cell function in both CRC and HCC, and inhibitors of either PGE_2_, or its downstream target indoleamine 2,3-dioxygenase (IDO) ablate the effect in culture (Balsamo et al., 2009; Li et al., 2012; Li et al., 2013; Bahrami et al., 2017). However, PGE_2_ does not appear to be a primary regulator in all cancer types, as CAFs derived from endometrial cancer are able to promote NK dysfunction independent of PGE_2_ signaling, and instead do so by downregulating cell surface poliovirus receptor (PVR/CD155), an important DNAM-1 ligand that is normally present on endometrial fibroblasts (Inoue et al., 2016).

CAF signaling can also act upon tumor cells to render them more resistant to NK-mediated killing. CAF-secreted matrix metalloproteinases (MMPs) decrease expression of MHC class I chain-related protein A and B (MICA/B) in tumor cells, which are important ligands for NK cells in recognizing distressed or malignant cells. Broad MMP inhibitors are able to ablate this effect (Ziani et al., 2017).

As NK cells become a major focus as candidates for novel off-the-shelf adoptive cell immunotherapies (Saetersmoen et al., 2019), they are likely to face many of the same barriers within the TME as T cells. Better understanding of the relationship between CAFs and NK cells will be vital going forward in order to give such therapies the best chance at therapeutic success.

### Mast cells

Mast cells can have varying effects on the TME. On one hand, mast cells drive a number of pro-tumorigenic processes in the TME, such as immunosuppression via expression of PD-L1 and promoting angiogenesis (Lv et al., 2019). On the other, mast cell infiltration into the TME is correlated with a positive prognosis across multiple cancers (Samoszuk et al., 2005).

CAFs are able to recruit mast cells to the TME via CXCL12/CXCR4 signaling (Figure 1) in prostate cancer but infiltrating mast cells were shown to be pro-tumorigenic in this setting (Ellem et al., 2014). Once recruited, mast cell signaling can enhance a variety of fibroblast functions including contraction of collagen lattices via stem cell factor (SCF)/c-kit signaling (Yamamoto et al., 2000) and increased production of collagenolytic enzymes via IL-1α and TNFα signaling (Dabbous et al., 1995). The broad and often conflicting functions of mast cells within the TME is an area in need of further study but given the close interactions of mast cells and fibroblasts, it is likely CAFs may be a major determinant in the role mast cells play.

### Neutrophils

As with many of the immune cell types mentioned above, neutrophils can have both antitumor and pro-tumor functions, although infiltration of tumor-associated neutrophils (TANs) correlates with negative outcome in many solid tumors (Ocana et al., 2017; Wu et al., 2019). In gastric cancer, TANs drive CAF formation by producing inflammatory cytokines like IL-17, IL-23, and TNF-α (Zhang et al., 2020). In pancreatic cancer, neutrophil extracellular traps (NETs) produced in response to pancreatic cancer metastases drive CAF formation in liver micrometastases, and treatment with DNase1 can significantly reduce CAF formation and metastasis growth in these models (Takesue et al., 2020).

On the flip side, CAFs can significantly influence neutrophils in the TME. Studies in breast cancer show that CAFs recruit neutrophils to the TME via IL-33 (Shani et al., 2020) and CXCL12 (Chen et al., 2019a). TANs recruited to the TME respond to CAF secreted IL-6 (Figure 1), as shown in both gastric cancer and HCC, which drives STAT3 and PD-L1 expression, allowing neutrophils to inhibit T cell function (Cheng et al., 2018; Zhu et al., 2014). Given that the role of neutrophils within the TME is only beginning to be understood at a mechanistic/molecular level, research directly connecting CAFs to neutrophils is still in early stages. However, neutrophils respond to many of the pathways already discussed in detail above and it is likely that they are an integral player in much of the CAF-immune crosstalk described.

## CAFs and cancer therapy

With the recent successes of immunotherapy in the treatment of cancer, obstacles to efficacy in the clinic have also emerged: (1) Immunotherapies work remarkably well in a subset of patients while being ineffective in many other patients, the reasons for which are still not completely understood and (2) some immunotherapies have struggled, particularly in highly stromagenic cancers such as pancreatic cancer, where almost no immunotherapeutic success has been realized. A greater understanding of how the tumor stroma and overall TME influence these therapies will provide important advances in these areas.

It is for these reasons that CAFs are a lucrative target for novel cancer therapies. Depletion of CAFs and targeting of CAF-dependent pathways can indirectly result in the death of malignant cells through both immune-dependent mechanisms and immune-independent mechanisms (Wang et al., 2014; Lo et al., 2015). Given that ECM dysregulation is one of the strongest predictors of failure in immunotherapies such as PD-L1 blockade (Chakravarthy et al., 2018), combinatorial approaches targeting immune cells and CAFs have been at the forefront. Another, arguably even more compelling, concept being developed is to use stroma-targeted therapies to make tumors more vulnerable to other types of therapies, including chemo-, tumor cell-targeted, and immuno-therapies.

**Figure 2.**
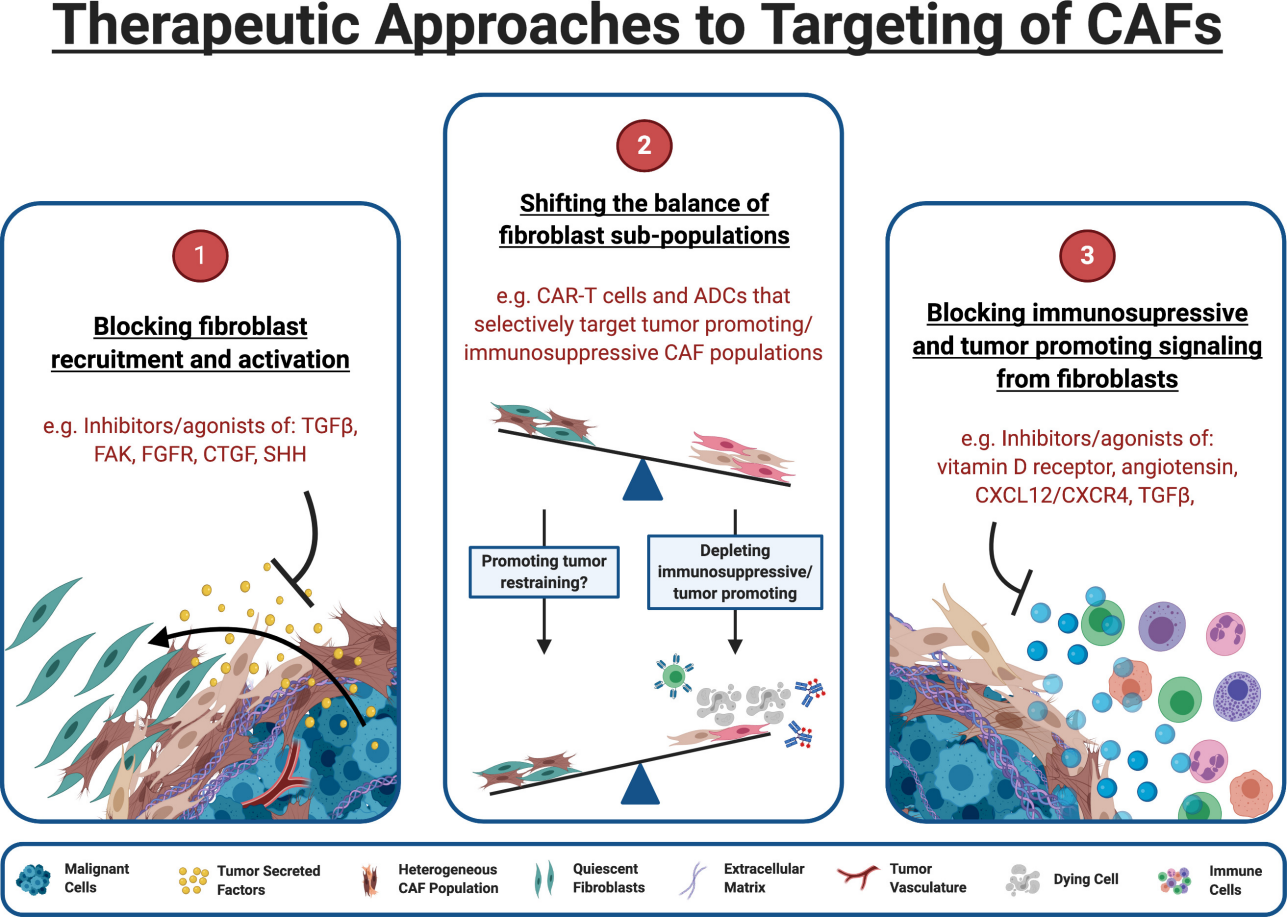
Therapeutic approaches to targeting of CAFs. (1) Inhibitors of pathways known to drive fibroblast activation can block tumor cells ability to manipulate fibroblasts for their own survival. (2) The functional heterogeneity between CAF populations in the TME means that targeting specific subpopulations can be an effective strategy. Targeted therapeutics such as chimeric antigen receptor (CAR) expressing T cells (CAR-T) and antibody-drug conjugates (ADCs) can target the fibroblast sub-populations responsible for tumor protection and immunosuppression while leaving quiescent and tumor restraining populations intact. (3) Blocking CAFs ability to exert immunosuppressive/tumor promoting influence within the TME may alleviate immunosuppression and allow immunotherapies to be effective within this space. Some targets, such as TGF-β, can act both upstream and downstream, blocking CAF formation and attenuating downstream signaling in CAFs that are already established. (Figure created with BioRender.com).

Several approaches are being taken to target fibroblasts: (1) targeting upstream pathways that drive fibroblast differentiation and reprogramming of CAFs, (2) taking advantage of the heterogeneity of the population in order to shift the influence of pro- versus anti-tumorigenic populations, and (3) targeting downstream effector pathways by which activated fibroblasts negatively influence the TME (Figure 2).

### Targeting specific CAF subpopulations

Early attempts to therapeutically target CAFs within the TME were unsuccessful in preclinical models and in patients, and actually worsened tumor prognosis (Özdemir et al., 2015; Rhim et al., 2014). These early attempts, however, were limited by an incomplete understanding of fibroblast heterogeneity and in retrospect may have selectively targeted populations of myofibroblast-like CAFs by specifically depleting SMA^+^ cells or targeting the sonic hedgehog pathway. These populations can exert tumor restraining activity in the particular cancer context studied (Özdemir et al., 2015; Rhim et al., 2014). In contrast, targeting other populations of fibroblasts have provided promising results.

One of the most successful approaches has been in targeting FAP^+^ fibroblasts (Cheng et al., 2002; Fearon, 2014). FAP^+^ cells can both promote tumor progression directly and present a barrier to immunotherapies through their production of ECM and direct signaling pathways (Puré and Blomberg, 2018; Lo et al., 2017; Lee et al., 2011). They are also major producers of certain immunosuppressive cytokines such as CXCL12 and CCL2 as mentioned earlier (Feig et al., 2013; Yang et al., 2016). As a result, depleting this population is thought to be an effective way to disrupt the desmoplasia and thus enhance immune infiltration, while either leaving tumor suppressing populations of fibroblasts undisturbed, or also resulting in depletion of such populations, but with the anti-tumorigenic depletion of the FAP^+^ cells proving dominant (Wang et al., 2014; Lo et al., 2015).

Indeed, multiple different approaches to depleting this population have shown success, from genetic depletion (Feig et al., 2013; Kraman et al., 2010), to using more translatable approaches like vaccines (Liao et al., 2009; Chen et al., 2015), drug delivering nanoparticles that are activated upon cleavage by FAP (Ji et al., 2016), FAP antibody-drug conjugates (ADCs) (Fabre et al., 2020), and CAR-T cells directed at FAP^+^ cells (Wang et al., 2014; Lo et al., 2015; Figure 2). Such treatments are typically designed to enhance the activity of conventional chemotherapies and immunotherapies but have also shown efficacy on their own, perhaps through favoring an immune permissive environment. So far, FAP^+^ populations have been the primary focus in stromal depletion therapies but as definition of CAF subpopulations are refined, we will likely see other targets taken advantage of.

### Targeting upstream and downstream pathways in CAFs

Of the therapies moving into, or already in the clinical space, there are two major therapeutic approaches being explored to target CAFs for cancer therapy. The first approach involves targeting with the upstream signaling required to drive fibroblasts from tumor restraining populations to tumor permissive or promoting (Figure 2). Drugs targeting these pathways currently include FAK inhibitors (Lin et al., 2018), Hedgehog inhibitors (Ko et al., 2016), fibroblast growth factor receptor (FGFR) inhibitors (Nishina et al., 2018), connective tissue growth factor (CTFG) antagonists (Neesse et al., 2013), and TGF-β inhibitors (de Gramont et al., 2017; Colak and ten Dijke, 2017). Targets such as TGF-β are multifaceted, influencing both CAF formation and downstream signaling of already established CAF populations.

The second approach involves targeting fibroblasts already transitioned to a tumor promoting/permissive state (Figure 2). These include therapies to reprogram activated CAFs to a more quiescent-like state, such as Vitamin D receptor agonists (Sherman et al., 2014) of which the drug paricalcitol has already entered phase I clinical trials (Fountzilas et al., 2018). Other therapies influence CAF matrix deposition, decompressing the tumor, normalizing vasculature, and thus conferring susceptibility to chemo- and immunotherapy. Examples include the angiotensin inhibitor losartan (Chauhan et al., 2013) and enzymatic therapeutics like PEGPH20, a pegylated hyaluronidase enzyme designed to degrade hyaluronic acid produced by fibroblasts in the TME. Hyaluronidase treatment showed promise in animal models and early clinical trials (Thompson et al., 2010; Hingorani et al., 2018). However, PEGHP20 in combination with nab-paclitaxel/gemcitabine failed to meet its primary endpoint of increased overall survival in Phase III clinical trials in pancreatic cancer patients and further development of the drug was halted (Hakim et al., 2019).

Treatments that target CAFs effector mechanisms that mediate their tumor promoting or immunosuppressive effects are also being developed. The RNA oligonucleotide drug NOX-A12 neutralizes CXCL12, inhibiting CXCR4 binding. NOX-A12 showed favorable responses in early clinical trials with pancreatic cancer and CRC and is continuing to be investigated clinically (NOXXON Pharma AG, 2019; NOXXON Pharma AG, 2010; NOXXON Pharma AG, 2017).

TGF-β is another downstream target with multiple attempts to target the pathway underway, including antisense oligonucleotides, neutralizing antibodies to TGF-β, and antibodies which block the TGF_-_β receptor TβRII (Colak and ten Dijke, 2017). However, many of these therapeutic strategies target TGF-β throughout the body and across multiple cell types. This could potentially limit efficacy seeing as TGF-β can be tumor suppressive under certain conditions (Moustakas and Heldin, 2016; Bhowmick et al., 2004). More directed approaches can sidestep TGF-β_’_s immunosuppressive effects possibly limiting off-target effects. For example, novel CAR-T cell therapies are being designed to maintain activation in the presence of TGF-β by co-expressing a dominant-negative TGF-βRII (Kloss et al., 2018).

It is becoming clear that fibroblasts play a major role in how immune cells respond to a variety of homeostatic perturbations and particularly cancer. Despite providing support for almost every major organ system in the human body, fibroblast biology has been overlooked in many disease processes. The recent push to understand their role in the context of tumor immunity and how their activation influences various biological systems will yield invaluable insight into how to improve cancer therapy and undoubtedly provide insight into many other disease processes as well.
